# Translating drug resistant tuberculosis treatment guidelines to reality in war-torn Kandahar, Afghanistan: A retrospective cohort study

**DOI:** 10.1371/journal.pone.0237787

**Published:** 2020-08-21

**Authors:** Anita Mesic, Waliullah H. Khan, Annick Lenglet, Lutgarde Lynen, Sadiqqulah Ishaq, Ei Hnin Hnin Phyu, Htay Thet Mar, Anthony Oraegbu, Mohammad Khaled Seddiq, Hashim Khan Amirzada, Jena Fernhout, Charity Kamau, Cono Ariti, Diana Gomez, Tom Decroo

**Affiliations:** 1 Médecins Sans Frontières, Amsterdam, The Netherlands; 2 Médecins Sans Frontières, Islamic Republic of Afghanistan, Kabul, Afghanistan; 3 Department of Medical Microbiology, Radboudumc, Nijmegen, The Netherlands; 4 Institute of Tropical Medicine Antwerp, Antwerp, Belgium; 5 Médecins Sans Frontières, Islamic Republic of Afghanistan, Kandahar, Afghanistan; 6 National Tuberculosis Control Programme, Islamic Republic of Afghanistan, Kabul, Afghanistan; 7 Centre for Trials Research, Cardiff University Medical School, Cardiff, United Kingdom; 8 Research Foundation Flanders, Brussels, Belgium; The University of Georgia, UNITED STATES

## Abstract

**Introduction:**

Afghanistan is affected by one of the world’s longest protracted armed conflicts, frequent natural disasters, disease outbreaks and large population movements and it suffers from a high burden of tuberculosis (TB), including rifampicin-resistant TB (RR-TB). The study shows Médecins Sans Frontières’ experiences with care for patients with RR-TB in Kandahar Province. We describe the uptake of RR-TB treatment, how World Health Organisation criteria for the choice between the short and an individualized regimen were implemented, and treatment outcomes.

**Methods:**

This is a retrospective cohort analysis of routinely collected data from RR-TB patients enrolled in care from 2016 until 2019. Descriptive analysis was performed to present characteristics of patients and treatment outcomes. Multivariable Cox analysis was performed to identify risk factors for having an unfavourable treatment outcome.

**Results:**

Out of 146 enrolled RR-TB patients, 112 (76.7%) started treatment: 41 (36.6%) and 71 (63.4%) with the short and individualized treatment regimen, respectively. Of 82 with results for fluoroquinolone susceptibility, 39 (47.6%) had fluoroquinolone-resistant TB. Seven patients with initially fluoroquinolone-resistant TB and three pregnant women started the short regimen and 18 patients eligible for the short regimen started the injectable-free individualized regimen. Overall, six-month smear and culture conversion were 98.7% and 97.1%, respectively; treatment success was 70.1%. Known initial fluoroquinolone resistance (aHR 3.77, 95%CI:1.53–9.27) but not choice of regimen predicted having an unfavourable outcome.

**Conclusion:**

Even though criteria for the choice of treatment regimen were not applied strictly, we have achieved acceptable outcomes in this cohort. To expand RR-TB care, treatment regimens should fit provision at primary health care level and take patient preferences into account.

## Introduction

Tuberculosis (TB) is the most important infectious disease in terms of global mortality and also a major cause of death related to antimicrobial resistance [1]. Access to care for persons with drug-resistant tuberculosis is limited and treatment outcomes in these patients are far from satisfactory. In 2018, there were 484,000 people with rifampicin-resistant (RR-TB), but only one-third was enrolled on treatment. Only 56% of those with RR-TB for whom outcomes were reported in 2018 were treated successfully [1].

Afghanistan has a population of over 37 million people [2] and is affected by one of the world’s longest protracted armed conflicts, frequent natural disasters, disease outbreaks and large population movements. In 2018, it was estimated that almost 2 million people were in need of humanitarian medical services [3]. The provision of medical care is hugely challenging, as exemplified by 85 registered attacks on health care facilities in 2018 [3]. Despite this challenging context, the National Tuberculosis Control Programme (NTP) decentralized TB services as part of the Basic Package of Health Services since 2005 [4].

In Afghanistan TB incidence is estimated at 189 per 100,000 population per year. About 3% (95%CI 1.4–5.3) and 12% (95%CI 11–14%) of new and retreatment cases have RR-TB, respectively. An estimated 2,500 (95%CI 1,000–4,700) patients developed RR-TB in Afghanistan in 2018 [5]. The diagnosis of RR-TB has increased since the roll-out of Xpert® MTB/RIF (Xpert; Cepheid, Sunnyvale, CA, USA), a rapid molecular test, now available in 68 sites in the country. Access to second-line line probe assay (SL-LPA; GenoType MTBDRsl; Hain Lifesciences, Nehren, Germany) testing is limited, as this test is not yet regularly performed at the National TB Reference Laboratory in Kabul. Patients diagnosed with RR-TB are usually treated with a standardized long (20-months or more) treatment regimen. RR-TB treatment is currently available in five provinces (Kabul, Balkh, Herat, Nangarhar, Kandahar). Barriers to care include lack of quality assured laboratory services, lack of qualified health staff at the health facility, insecurity, cultural barriers, and TB related stigma [3].

Since 2016, in Kandahar, Médecins Sans Frontières (MSF) used either a short treatment regimen (STR), as recommended in the 2016 WHO Guidelines [6] and described in the national guideline [7], or an individualized long regimen, taking into account drug susceptibility testing (DST) results, previous exposure to TB drugs, age, concomitant treatment and comorbidities. We adapted 2016 WHO criteria to decide if a patient would be treated with either the short or long RR-TB treatment regimen. Previously analyses used data on TB drug resistance prevalence to calculate the proportion of patients eligible for the STR, which ranged between 10 and 51% [8–13]. However, since the 2016 WHO criteria were published, no previous study described in detail how these criteria were translated to the operational reality of a TB programme. We therefore describe characteristics and treatment outcomes of patients started on RR-TB treatment, by regimen in Kandahar since 2016. Moreover, we estimate predictors for having an unfavourable treatment outcome.

## Methods

### Design and study population

This is a retrospective cohort study of all patients started on RR-TB treatment in Kandahar, Afghanistan, between 22^nd^ October 2016 and 18^th^ November 2019. The study observation period ended on 20^th^ March 2020.

### Study setting

Kandahar province has a population of approximately 1,200,000 people. Due to conflict and insecurity health care actors are sparse. In close collaboration with the Kandahar Provincial Health Directorate (PHD) and the NTP, MSF supports diagnosis and treatment of patients with drug-sensitive TB in the Regional Merwaiz Hospital and Provincial TB Center Kandahar. Patients diagnosed with RR-TB on Xpert MTB/RIF are referred to the MSF RR-TB facility.

### Bacteriological investigations

From patients with RR-TB, specimens are collected before treatment initiation and sent to the Institute of Tropical Medicine Antwerp in Belgium (ITM; Supra-National Reference Tuberculosis Laboratory), where SL-LPA together with phenotypic resistance testing are performed. Results of molecular testing are obtained after about three weeks. During treatment, monthly specimens are collected for smear microscopy performed in Kandahar and culture at the ITM.

### Choice of treatment regimen

Patients should start treatment within one week after RR-TB diagnosis. Exclusion criteria for the STR were adapted from the 2016 WHO criteria and were: known initial resistance to fluoroquinolones, more than one month previous exposure to second-line TB drugs, age below 15 years (limited capacity to perform audiometry in children), pregnancy or having a medical contraindication to any of the drugs in the regimen. The patient’s preference for either the individualized or short regimen was also taken into account. In contrast with the 2016 WHO criteria, initial resistance to drugs other than fluoroquinolone was not an exclusion criterium for using the STR [6]. While waiting for the results of SL-LPA testing, those without exclusion criteria were initiated on the STR. When SL-LPA results arrived and showed resistance to either fluoroquinolone or injectables, the treatment regimen was individualized. Those not eligible, not preferring or not tolerating the STR received the individualized (20+) long regimen following the national and WHO guidelines, including new (bedaquiline, delamanid) and repurposed drugs (linezolid, clofazimine, imipenem).

### Clinical management and follow-up

Ambulatory treatment was preferred and only clinically unstable patients were hospitalised at the MSF RR-TB facility. Daily administration of second-line injectables and monthly follow-up visits were organised at the outpatient department of the same facility. For almost all patients, oral treatment was self-administered (SAT), and supported by a family member or other caretaker. For patients coming from outside of Kandahar city MSF provided accommodation, which could be utilized throughout their treatment, during the intensive phase with daily administration of injections, or for a short stay during monthly follow-up visits. All travel expenses for patients and their caretakers were reimbursed by MSF. We systematically monitored patients for adverse events (AE), which were managed based on the EndTB clinical guidelines [14]. Severe adverse events (SAE) were reported to the national programme as part of the Active Tuberculosis Drug-Safety Monitoring and Management (aDSM) guidance from the WHO [15]. Psychosocial support was part of the care package and included counselling sessions by trained counsellors and participation in a peer support group during follow-up visits in the clinic. For patients on SAT who had access to a telephone, weekly phone calls with the counsellor were organized to provide adherence support and to monitor adverse events. Systematically post-treatment monitoring was done for a duration of one year, including a clinical assessment and bacteriological and radiological investigations in those with symptoms.

### Contact tracing

All close household contacts were screened for the presence of TB symptoms. In addition, all children under the age of five and all symptomatic contacts had a chest radiography and were asked to provide a sputum sample for bacteriological analysis. Clinical assessment of contacts was done at the start of treatment of the index case and then every 3 months. Travel expenses for contacts were reimbursed.

### Data collection and analysis

The study used routine programme data collected from standardized patient forms and retrieved from the MSF TB programme database (named *Koch6*). The dataset was exported into statistical software *STATA* (version 14.2, Texas, USA) for statistical analysis. Independent variables included age, gender, marital status, employment, province of origin, radiological severity, case definition, body mass index (BMI), co-morbidities, drug-resistance, and treatment regimen. Case and treatment outcome definitions followed the WHO recommendations [16]. Death, lost to follow-up (LFU) or treatment failure were considered as unfavourable treatment outcomes.

All patients that initiated RR-TB treatment were included in the descriptive analysis. Categorical variables were summarized using frequencies and percentages. Continuous variables were summarized using medians and interquartile ranges (IQRs). BMI (<18.5 kg/m2, ≥1 18.5 kg/m2) and age (≤15, >15 years) were categorized. Categorical variables marital status (with a partner/without a partner) and employment (employed/not employed) were dichotomized. Radiological severity on chest radiography included the following categories: normal (no pathological changes detected), non-severe (unilateral pathological changes detected but no cavities present) and severe (cavity(s) and/or bilateral disease). The chi-squared test was used to assess whether baseline characteristics were associated with pre-treatment attrition (either LFU or death). The chi-squared test was used to assess differences in proportions between both cohorts (STR vs individualized regimen). Patients with a treatment outcome were included in the Cox univariate and multivariate regression analysis. Unadjusted hazard ratios (HR) with their 95%CI were calculated to assess if independent variables were associated with having an unfavourable outcome. Independent variables with p<0.10 in univariate analysis and potential confounders (province of origin, regimen) were included in the final multivariable model regardless of the p-value. In patients with a positive initial smear/culture result and who started treatment at least 4 months before the end of the study period Kaplan Meier survival curves were plotted to show time to smear/culture conversion by regimen. The log-rank test was used to test significance of differences between strata according to the regimen.

### Ethical approval

The study was approved by the Institutional Review Board, Ministry of Public Health Afghanistan (A07190052) and fulfilled the exemption criteria set by the MSF independent Ethical Review Board [17] for a posteriori analyses of routinely collected clinical data and thus did not require MSF ERB review. It was conducted with permission from the MSF Medical Director, Operational Center Amsterdam. All data used for the study originated from the medical records of the MSF RR-TB facility and were fully anonymized before making them available for the analysis. Data were analysed between20 March and 15 May 2020.

## Results

### Enrolment on RR-TB treatment

**Fig 1:**
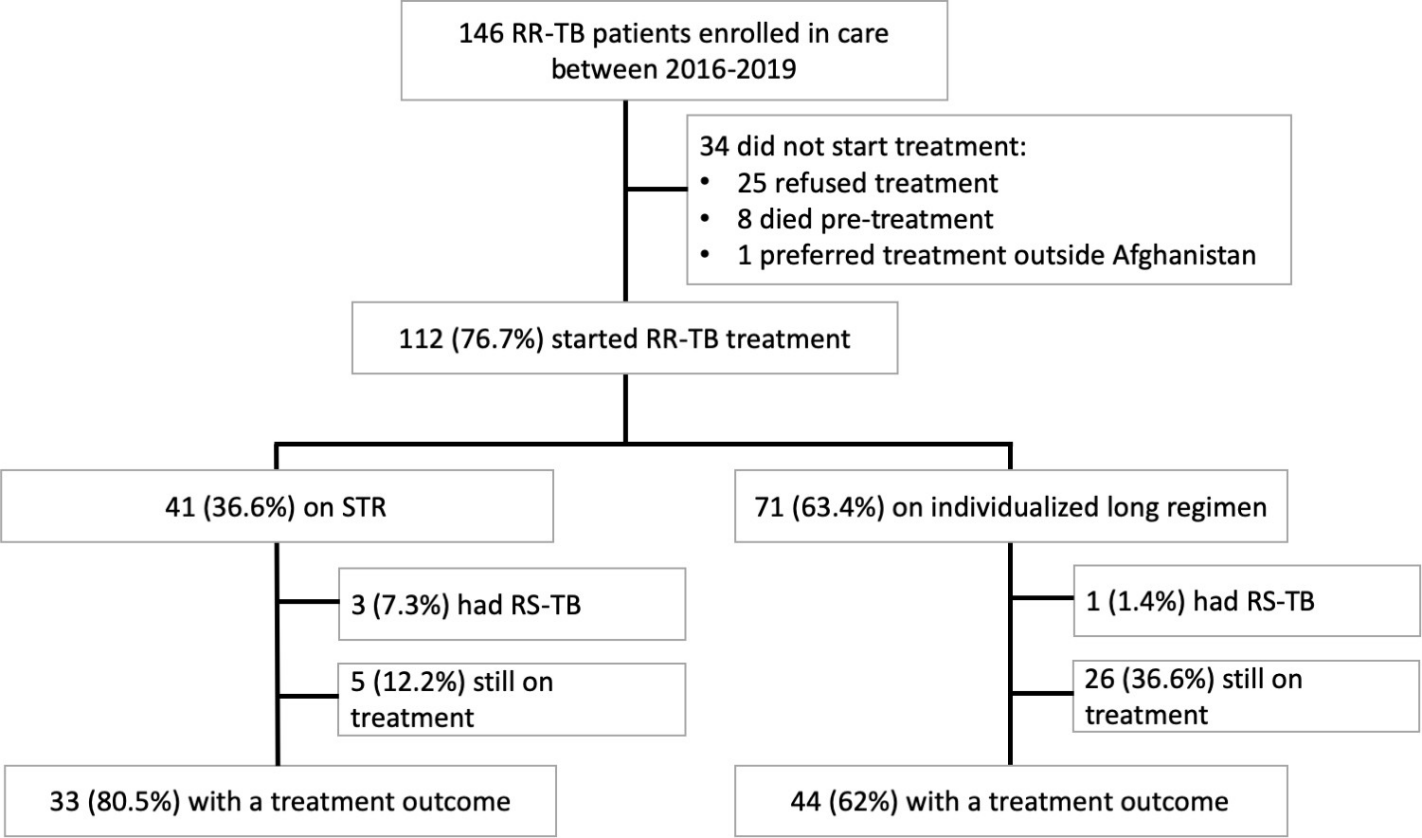
Kandahar cohort flowchart.

During the study period (2016–2019) 146 RR-TB patients enrolled in care at the MSF RR-TB facility (Fig 1). Of the 146 patients, 112 (76.7%) started treatment during the study period. Of the 34 (30.3%) patients that did not start on treatment, 25 (73.5%) refused treatment, 8 (23.5%) died before starting treatment and one (3.0%) expressed preference for treatment outside Afghanistan.

Similar proportions of female and male patients started treatment (77.3% (68/88) in females vs. 75.9% (44/58) in males). Similar proportions of patients from both inside and outside of Kandahar province started treatment (75.3% (67/89) from outside Kandahar vs 78.9% (45/57) from inside Kandahar). All children (n = 16) diagnosed with RR-TB were initiated on treatment. Treatment initiation was more likely in patients that reported contact with an existing RR-TB case (96.0% (24/25) with contact vs. 72.7% (88/121) without contact history; p = 0.02). Patients with a positive baseline smear result were also more likely to start the treatment compared to those with a negative smear (80.9% (89/110) vs. 60.0% (18/30); p = 0.02). Patients with a positive baseline culture result were more likely to start the treatment compared to those with a negative culture (93.1% (81/87) vs. 70.3% (26/37); p<0.01).

### Baseline characteristics

Among 112 patients that started on treatment, 67 (59.8%) originated from outside Kandahar province, 68 (60.7%) were female; and the median age was 32.2 years (IQR 21.1–39.7). Twenty-four patients were diagnosed after contact tracing (21.5%), including five (63%) of eight patients under five years of age. About half of the patients (48.2%) were not previously treated for TB. The baseline demographic characteristics were similar for patients started on STR and individualized treatment regimen (Table 1).

**Table 1:** Demographic characteristics of patients enrolled in RR-TB care, by treatment.

Characteristic	Treatment not started (n = 34)		Treatment started (n = 112)				
			Short treatment regimen (n = 41)		Individualized regimen (n = 71)		p-Value^a^
	N	%	N	%	N	%	
**Geographical origin**							0.07
Kandahar province	12	35.3	21	51.2	24	33.8	
Another province	22	64.7	20	48.8	47	66.2	
**Gender**							0.24
Male	14	41.2	19	46.3	25	35.2	
Female	20	58.8	22	53.7	46	64.8	
**Age groups**							0.63
Age <15	0		5	12.2	11	11.5	
Age = > 15	34	100.0	36	87.8	60	84.5	
**Marital Status**							0.11
Married or living with somebody	16	47.1	32	78.1	46	64.8	
Single	3	8.8	8	19.5	24	33.8	
Missing	15	44.1	1	2.4	1	1.4	
**Employment Status**							0.31
Employed	0		3	7.3	2	2.8	
Non-employed	16	47.1	34	82.9	57	80.3	
Missing	18	52.9	4	9.8	12	16.9	
**Contact history**							0.70
Diagnosed as a contact	1	2.9	8	19.5	16	22.5	
Not diagnosed as a contact	33	97.1	33	80.5	55	77.5	

^a^The chi-squared test was used to compare categorical variables by type of regimen, among those started on treatment

Of the 112 patients that started on treatment, 68 (60.7%) were undernourished when starting treatment, 95 (84.2%) had severe disease on chest radiography, 62 (55.3%) had cavitary lung disease, 89 (79.5%) were smear-positive and 81 (72.3%) were culture-positive at baseline. Only 3 patients had extrapulmonary TB (EPTB: two with meningitis and one with osteoarticular EPTB. Of 93 (83.0% of 112) with a baseline hearing status recorded, 49 (52.6%) had some hearing loss before starting RR-TB treatment. Baseline disease characteristics were similar for both treatment regimen cohorts (Table 2).

**Table 2:** Disease characteristic among patients who started RR-TB treatment.

	Treatment not started (n = 34)		Treatment started (n = 112)				
			Short treatment regimen (n = 41)		Individualized regimen (n = 71)		p-Value^a^
	N	%	N	%	N	%	
**Case definition**							0.06
New	21	61.8	15	36.6	39	54.9	
Previously treated	13	38.2	26	63.4	32	45.1	
**Previous TB regimen**							0.02
First line drugs	18	52.9	22	46.3	28	39.4	
Second line drugs	0	0	0	0	3	4.2	
Unknown (missing)	16	47.1	19	46.3	40	56.3	
**BMI**							0.17
< 18.5 kg/m2	2	5.9	22	53.7	46	64.8	
> = 18.5 kg/m2	7	20.6	19	46.3	23	32.4	
Unknown (missing)	25	73.5	0		2	2.8	
**Site of TB infection**							0.90
Pulmonary	34	100.0	40	97.6	69	97.2	
Extrapulmonary	0		1	2.4	2	2.8	
**Baseline smear**							0.06
Positive	21	61.7	36	87.8	53	75.7	
Negative	12	35.3	3	7.3	15	21.3	
Unknown (missing)	1	2.9	2	4.9	3	4.23	
**Baseline culture**							0.48
Positive	6	17.7	31	75.6	50	70.4	
Negative	11	32.3	8	19.5	18	25.4	
Unknown (missing)	17	50.0	2	4.9	3	4.2	
**Baseline symptoms**							0.44
Symptomatic	13	38.2	41	100.0	70	98.6	
Non symptomatic	21	61.8	0		1	1.4	
**Baseline Hearing loss**^b^							0.85
No	NA^1^	NA	18	43.9	26	36.6	
Yes	NA	NA	21	51.2	28	39.4	
Unknown (missing)	NA	NA	2	4.9	17	24.0	
**Extent of disease on chest radiograph**							0.67
Severe	5	14.7	34	82.9	61	85.9	
Non severe or normal	29	85.3	7	17.1	10	14.1	
**Pregnancy**							0.72
No	19	95	19	86.4	42	91.3	
Yes	1	5.0	3	13.6	4	8.7	
**Diabetes**							0.12
No	33	97.1	41	100.0	67	94.4	
Yes	1	2.9	0		4	5.6	
**Cardiovascular disease**							0.12
No	33	97.1	41	100.0	67	94.4	
Yes	1	2.9	0		4	5.6	

^a^The chi-squared test was used to compare categorical variables by type of regimen among those that started treatment.

^b^Hearing assessment was done after the patient agreed to start the treatment

Baseline isoniazid DST results were missing for 28 (25%) patients. Of 81 (72.3%) with LPA results, katG and inhA mutations were identified in 33 (40.7%) and 8 (9.9%) of patients, respectively, and only 2 (2.4%) patients had mutations in both genes. Of 43 (38.4%) patients with baseline pyrazinamide DST results, 20 (46.5%) had TB resistant to pyrazinamide. The frequency of initial resistance to isoniazid and pyrazinamide was similar for both treatment regimen cohorts (Table 3). Of 82 patients with fluoroquinolone DST results, 39 (47.6%) had initially fluoroquinolone-resistant TB. Of these 39 patients, 32 (82.1%) were treated with the individualized regimen. The proportion with initial fluoroquinolone-resistant TB was higher among patients started on the individualized regimen (60.4% (32/53) vs 24.1% (7/29); p = 0.002). Of 75 patients with second-line injectable DST results, 3 (4.0%) had TB resistant to second-line injectables. All three patients were treated with the individualized regimen.

**Table 3:** Resistance pattern among patients who initiated treatment.

	Short treatment regimen (n = 41)		Individualized regimen (n = 71)	
	N	%	N	%
**Resistance to isoniazid**				
Resistant	14	45.2	32	60.4
*katG*^a^ mutation	11	35.5	22	41.5
*inhA*^a^ mutation	1	3.2	7	13.2
*katG* and *inhA* mutation	0	0	2	3.8
Phenotypic resistance (only)	2	6.5	1	1.9
Sensitive or wild type	17	54.8	21	39.6
Result missing	10	24.4	18	25.4
**Resistance to pyrazinamide**				
Resistant	9	52.9	11	42.3
*pncA*^a^ sequencing mutation	3	17.6	3	11.6
Phenotypic resistance (only)	6	35.3	8	30.7
Sensitive or wild type	8	47.1	15	57.7
Result missing	24	58.5	45	63.4
**Resistance to fluoroquinolones**				
Resistant	7	24.1	32	60.4
*gyrA*^a^	7	24.1	24	45.3
*gyrB*^a^	0		1	1.9
*gyrA + gyrB*	0		2	3.8
Phenotypic resistance (only)	0		1	1.9
Sequencing resistance (only)	0		4	7.5
Sensitive or wild type	22	75.9	21	39.6
Result missing	12	29.3	18	25.4
**Resistance to second line injectables**				
Resistant	0		3	6.4
*Rrs*^a^ mutation	0		3	6.4
Phenotypic resistance(only)	0		0	
Sensitive or wild type	28	68.3	44	93.6
Result missing	13	31.7	24	33.8

^a^ KatG, InhA, pncA, GyrA, GyrB, rrs are specific bacillary’ genes, which mutations confer resistance to particular tuberculosis drugs

### Application of adapted WHO 2016 criteria for choice of regimen

Of 112 patients that started treatment, 41 (36.6%) and 71 (63.4%) were treated with the short and individualized regimen, respectively. The reasons provided (one reason per patient) for starting the individualized regimen included: baseline hearing loss (20 patients, 28.2%), being younger than 15 years (11 patients, 5.5%), pregnancy (4 patients, 5.6%), refusal to start treatment with second-line injectables due to access issues (18 patients, 25.4%), previous treatment with second-line TB drugs for longer than one month (3 patients, 4.2%), and initial fluoroquinolone resistance with no other reason (15 patients, 21.1%). Among patients with initially fluoroquinolone-resistant TB, seven were treated with the STR. Three women in late-stage pregnancy preferred the STR. STR was also started in 15 (36.6%), five (12.2%), and one (2.4%) patient presented with mild, moderate or severe hearing impairment respectively, as registered on their baseline audiometry. All patients on the individualized regimen were treated according to national and international (WHO) guidelines and received at least 4 likely active TB drugs. The composition of the individualised regimen is shown in Table 4.

**Table 4:** Drugs included in individualized regimens.

Drug	N	%
Bedaquilline	23	32.4
Delamanid	16	11.0
Clofazimine	55	77.5
Moxifloxacine	40	56.3
Levofloxacine	17	23.9
PAS	7	9.9
Cycloserine	28	34.4
Ethionamide	47	66.2
Linezolid	38	53.5
Capreomycin	2	2.8

### Tolerability

Table 5 reports frequency of AE episodes. Of patients on the STR and the individualized regimen, 15 (36.6%) and 31 (44.3%) had at least one AE reported (p = 0.46). In these 46 patients, 124 AE episodes were recorded: 61 (49.2%) were mild, 49 (39.5%) moderate and 14 (11.3%) severe. None of AE was life-threatening. Fourteen severe AE were due to: hepatotoxicity (4), peripheral neuropathy (6), anemia (1), thrombocytopenia (1), hearing loss (1), and gastrointestinal toxicity (1). The culprit drugs for hepatoxicity were pyrazinamide (two patients), high-dose isoniazid (one patient) and bedaquiline (one patient). Linezolid caused all six episodes of severe peripheral neuropathy as well as one episode of severe anemia. Culprit drugs were stopped and regimens adapted. No patient had to definitively stop treatment and in none more than one drug was changed. In patients with AE and without AE, 6 (26.1%) and 17 (73.9%) had an unfavorable treatment outcome (p = 0.01), respectively. One patient suffered from idiopathic thrombocytopenia while in another patient gastrointestinal symptomatology was related to food poisoning.

**Table 5:** Typology and grading of 124 reported adverse events.

	Short treatment regimen					Individualized regimen				
	Mild	Moderate	Severe	Total		Mild	Moderate	Severe	Total	
				N	%				N	%
Dermatological	1	1	0	2	5.7	8	4	0	12	13.5
Endocrine or metabolic	4	0	0	4	11.4	0	0	0	0	0
Gastrointestinal	7	5	0	12	34.3	15	6	1	22	24.7
Hematological	0	0	1	1	2.9	1	2	1	4	4.5
Hepatic	6	5	2	13	37.1	0	3	2	5	5.6
Musculoskeletal	0	0	0	0	0	6	1	0	7	7.9
Renal	0	0	0	0	0	0	4	0	4	4.5
Neurological	0	1	0	1	2.9	8	8	6	22	24.7
Hearing loss	0	0	1	1	2.9	1	3	0	4	4.5
Systemic hypersensitivity	1	0	0	1	2.9	1	1	0	2	2.2
Cardiological (QTFc)^a^	0	0	0	0	0	2	5	0	7	7.9
**Total**	19	12	4	35	100.0	42	35	10	89	100.0

^a^Corrected QT interval considered abnormal if > 500 ms or >60 ms increase from the baseline value

### Treatment response

**Fig 2:**
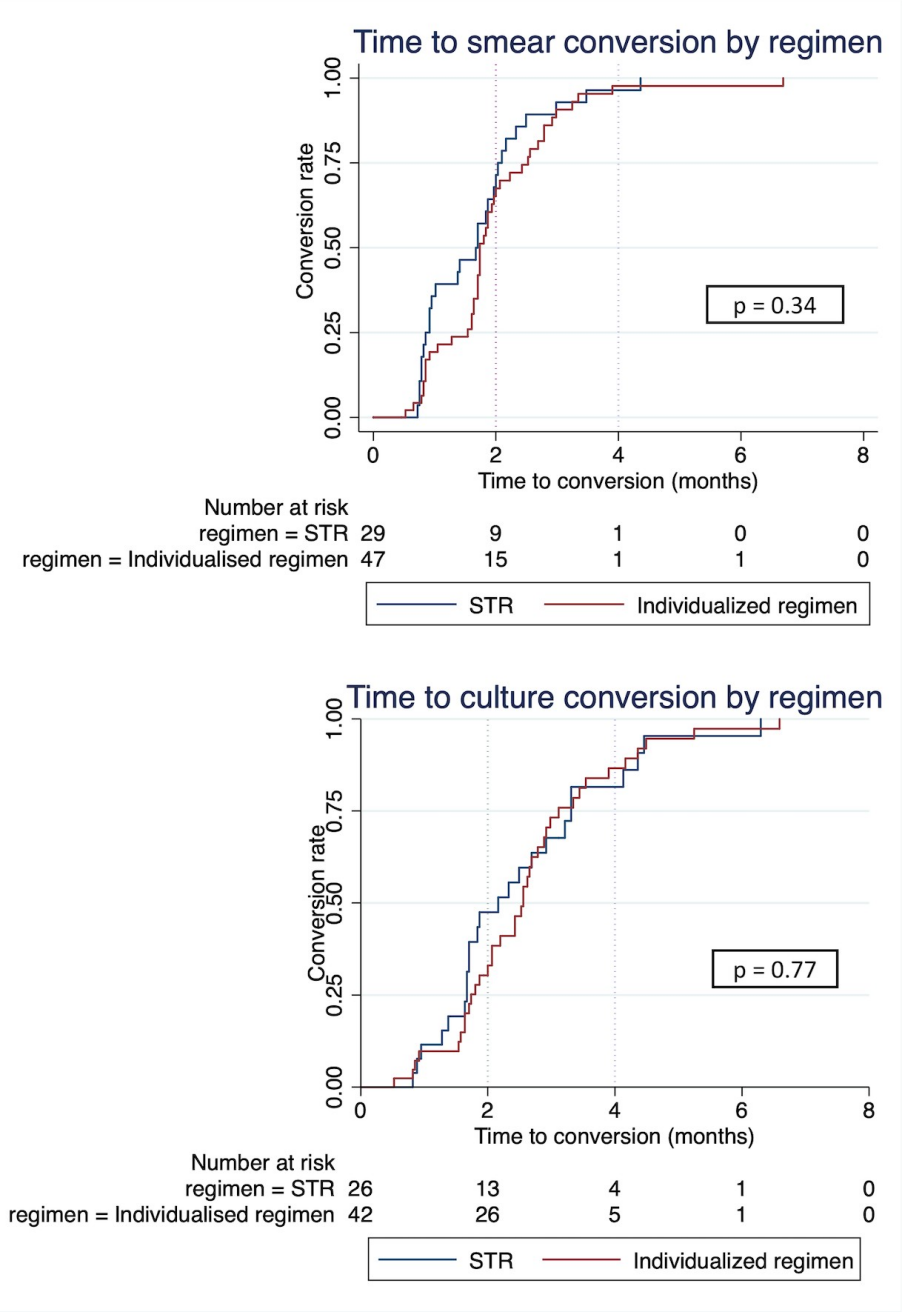
Kaplan-Meier estimates of time to smear and culture conversion. Smear and culture conversion are considered as at least one negative result following positive baseline results and not followed by smear or culture reversion during treatment.

Four patients on RR-TB treatment were transferred to the drug-sensitive TB programme because results from the reference laboratory showed rifampicin-susceptible TB on FL-LPA and Xpert MTB/RIF (genome sequencing ongoing). Their outcomes were not included in the analysis. Of the remaining, RR-TB treatment outcomes were known for 77 (71.3%) patients, while 31 (28.7%) patients were still on treatment at the end of the study period. In patients on the STR and the individualized regimen, four-month smear conversion was achieved in 28 (96.6%) and 32 (97.9%), respectively, and six-month smear conversion in 29 (100%) and 46 (97.9%), respectively (Fig 2). In patients on STR and individualized regimen, four-month culture conversion was achieved in 22 (84.6%) and 37 (88.1%), and six-month culture conversion in 25 (96.2%) and 41 (97.6%), respectively. The median time to smear conversion was 51.5 days (IQR 27–63) for the STR and 53 days (IQR 43–75.5) for the individualized regimen. The median time to culture conversion was 61.5 days (IQR 50.5–99.5) for the STR and 77.5 days (IQR 53–95) for the individualized regimen. Time to smear conversion (p = 0.34) or culture conversion (p = 0.77) was similar for both regimens (Fig 2).

Favorable treatment outcomes were achieved among 79.8% and 63.6% in patients receiving the STR and the individualized regimen, respectively (Table 6). Eleven (14.3%) patients died: five died due to a co-morbidity, four died after stopping treatment but before being declared LFU, one died due to TB meningitis, and for five patient the cause of death was not recorded. No patient died due to AE. Excluding those LFU, mortality was more frequent in patients treated with the individualized regimen (26.3% 10/38 vs 3.7% (1/27); p = 0.02) No patient developed treatment failure. Among seven patients treated with the STR and with initially fluoroquinolone-resistant TB three (49.9%) were cured, one died (14.3%) and three (49.9%) were LFU. All three pregnant women treated with the STR were cured and were without complications among the mothers or their infants.

**Table 6:** Treatment outcome (n = 77).

Regimen	Short treatment regimen (n = 33)^a^		Individualized regimen (n = 44)^b^	
Treatment outcome	N	%	N	%
Cured	26	79.8	21	47.7
Completed	0	0.0	7	15.9
Death	1	3.0	10	22.7
Treatment failure	0	0.0	0	0.0
Lost to follow-up	6	18.2	6	13.6
Transfer out	0	0.0	0	0.0

^a^At the time of analysis 5 patients were still on treatment and 3 patients were excluded from DR-TB cohort (and treatment) due to discrepancy in rifampicin resistance test results

^b^At the time of analysis 26 patients were still on treatment and 1 patient was excluded from DR-TB cohort (and treatment) due to discrepancy in rifampicin resistance test results

Patients with initial resistance to fluoroquinolones and/or SLIJ had an almost four times higher risk of experiencing an unfavorable outcome (vs susceptible to both or without DST result; aHR 3.77, 95%CI 1.53–9.27; p = 0.004). Patients with severe disease on chest radiography at baseline were 77% less at risk of having an unfavourable outcome (versus normal or non-severe disease; aHR 0.23, 95%CI 0.08–068; p = 0.002). The choice of treatment regimen (aHR 1.14, 95%CI 0.42–3.06; p = 0.79) was not correlated with having an unfavorable outcome (Table 7).

**Table 7:** Associations of participants characteristics with favourable and unfavourable outcomes (death and lost to follow-up).

Exposure variables	Total	Favorable Outcomes	Unfavorable outcomes	HR (95%CI)^a^	p-value	AHR^b^	p-value
						(95% CI)	
						(N = 67)	
**District**							
**Kandahar**	40 (52%)	31 (57.4%)	9 (39.1%)	0.58 (0.25–1.35)	0.21	0.82 (0.34–2.02)	0.67
**Other**	37 (48%)	23 (42.6%)	14 (60.9%)		Ref		
**Age (years)**							
**< 15 years**	11 (14.3%)	8 (14.8%)	3 (13%)	Ref	Ref	NA	NA
**> = 15 years**	66 (85.7%)	46 (85.2%)	20 (87%)	0.99 (0.29–3.35)	0.99		
**Gender**							
**Male**	34 (44.2%)	24 (44.4%)	10 (43.5%)	Ref	Ref	NA	NA
**Female**	43 (55.8%)	30 (55.6%)	13 (56.5%)	0.97 (0.43–2.23)	0.96		
**Marital status**							
**With partner**	55 (71.4%)	41 (75.9%)	14 (60.9%)	Ref	Ref	NA	NA
**No partner**	22 (28.6%)	13 (24.1%)	9 (39.1%)	1.73 (0.75–4.01)	0.20		
**Employment**							
**Employed**	4 (5.2%)	2 (3.7%)	2 (8.7%)	Ref			
**Unemployed**	69 (89.6%)	51 (94.4%)	18 (78.3%)	0.31 (0.07–1.33)	0.11	NA	NA
**Missing**	4 (4.2%)	1 (1.9%)	3 (13%)	NA			
**Previous treatment**							
**New**	42 (54.5%)	28 (51.9%)	14 (60.9%)	0.66 (0.28–1.54)	0.34	NA	NA
**Previously treated**	35 (45.5%)	26 (48.1%)	9 (39.1%)	Ref			
**Regimen**							
**Short Course**	33 (42.9%)	26 (48.2%)	7 (30.4%)	Ref			
**Individualized**	44 (57.1%)	28 (51.8%)	16 (69.6%)	1.32 (0.52–3.36)	0.56	1.14 (0.42–3.06)	0.79
**Radiological severity**							
**Normal/not severe**	11 (14.3%)	5 (9.3%)	6 (26.1%)	Ref	Ref	Ref	0.007
**Severe**	66 (85.7%)	49 (90.7%)	17 (73.9%)	0.33 (0.13–0.85)	0.02	0.23 (0.08–0.68)	
**BMI (kg/m2) (n = 73)**							
**< 18.5**	28 (36.4%)	21 (38.9%)	7 (30.4%)	Ref	Ref	NA	NA
**> = 18.5**	47 (61%)	31 (57.4%)	16 (69.6%)	1.36 (0.56–3.32)	0.45		
**Missing**	2 (2.6%)	2 (3.7%)	NA				
**Baseline smear**							
**Negative/no result**	8 (10.4%)	5 (9.3%)	3 (13%)	Ref	Ref	NA	NA
**Positive**	69 (89.6%)	49 (90.7%)	20 (87%)	0.59 (0.18–2.03)	0.60		
**Baseline culture**							
**Negative/no result**	15 (19.5%)	10 (18.5%)	5 (21.7%)	Ref	Ref	NA	NA
**Positive**	62 (80.5%)	44 (81.5%)	18 (78.3%)	0.71 (0.26–1.91)	0.49		
**Diabetes**							
**No**	2 (2.9%)	53 (98.2%)	22 (95.7%)	Ref	Ref	NA	NA
**Yes**	68 (97.1%)	1 (1.9%)	1 (4.3%)	2.22 (0.30–16.61)	0.46		
**Resistance to fluoroquinolone**^c^							
**No**	28 (48.3%)	25 (60.9%)	3 (17.7%)	Ref		Ref	
**Yes**	30 (51.7%)	16 (39.1%)	14 (82.3%)	4.2 (1.19–14.6)	0.03	3.77 (1.53–9.27)	0.004

^a^ Hazard ratio (univariate Cox analysis)

^b^ Adjusted Hazard Ratio (adjusted for all variables listed in the table)

^c^ There were three patients with concomitant resistance of fluoroquinolone and SLIJ and none of the patients had isolated SLIJ resistance

## Discussion

This study describes how WHO criteria for enrolment on either the STR or the individualized long regimen were translated to the reality of the RR-TB program in Kandahar, Afghanistan. Among 112 patients who started treatment 41 (36.6%) were on the STR while 71 (63.4%) received an individualized long regimen. We used adapted WHO criteria for STR eligibility in a flexible manner [6]. To not delay treatment while waiting for fluoroquinolone DST results, patients were offered to start the STR if they had no other exclusion criteria. If DST results were not obtained, due to logistical constraints or an inadequate quality of the sputum sample, STR exclusion criteria could not be fully assessed. Moreover, patient’s preferences and the interplay between different clinical criteria were considered. We treated ten patients with STR which would have been excluded from this regimen if WHO criteria would have been used more strictly (seven with fluoroquinolone resistance and three pregnant women). Also, a large proportion of patients treated with the second-line injectable containing STR had baseline hearing impairment. They were treated with the STR either because impairment was mild with conductive hearing loss, or because patients insisted to be treated for a shorter duration. On the other hand, of 71 treated with the individualized regimen, 18 (25.3%) were eligible for the STR but preferred the injectable-free long regimen. They were unable to commit to daily clinic visits for injectables administration. Community-based provision of TB care, including the administration of injectables, has proven to be effective and acceptable in other settings [18, 19] but was not feasible in our setting as it was no safe to travel outside the city limits of Kandahar. Our consideration for patient’s preferences when choosing a regimen is coherent with the 2019 WHO recommendation [20]. If we would used the WHO criteria more strictly, only 33 (29.5%) patients would be treated with the STR. The choice corresponded in 65 (58%) patients with the WHO criteria. Besides medical criteria also the patients’ preferences, logistics and security constraints had to be taken into account.

Despite a high prevalence of fluoroquinolone resistance (47.6%) in our study population, comparable to 52.1% reported in neighboring Baluchistan (Pakistan) [21], 70.1% of patients were treated successfully. Treatment success was 79.8% in patients treated with the STR. Globally the success rate among patients treated for RR-TB is 55% [1], while the WHO target for RR-TB treatment success is set at >75% [1]. We reported 47.7% treatment success in patients with fluoroquinolone-resistant TB and treated with the individualized regimen. Globally the success rate for extensively resistant TB is 34% [1]. The proportion LFU was 18.2% and 13.6% in those treated with the STR and individualized regimen, respectively. This contrasts with findings from the recent meta-analysis, which showed that patients treated with the STR were less likely to be LFU (4.2% vs 14.6% in those treated with an individualized regimen) [22]. More than half of our patients came from outside Kandahar. Besides the requirement of daily clinic visits for the administration of an injection, it is likely that contextual factors and cultural barriers contributed to high LFU rates in war-torn Kandahar [23]. Mortality was higher among those treated with the individualized regimen, which is probably explained by the much higher frequency of baseline fluoroquinolone resistance in this cohort and concurrent co-morbidities. Indeed, initial fluoroquinolone resistance was a strong predictor of having an unfavorable outcome, which is coherent with other studies [10, 21, 26–30]. After adjusting for initial fluoroquinolone resistance, severity of disease on chest radiography, and other confounders, the choice of regimen was not correlated with having an unfavorable outcome. However, our sample size might not have been large enough to detect a difference if there was one. Moreover, our study was not designed to compare the effectiveness of both regimens.

Both regimens were bacteriologically very effective. No patient developed treatment failure. Many patients had one or more known risk factors for delayed conversion [8, 24–27]. Despite 47.6% initially fluoroquinolone-resistant TB, a poor nutrition status in about half of our patients, and over 80% with advanced disease on chest radiography, almost all patients achieved six-month smear conversion. Six-month culture conversion was similarly high in both treatment regimens. Our findings compare well with those from other studies, reporting 6-month conversion rates between 87%-98% [8, 24–27].

Overall, RR-TB treatment was well tolerated and the frequency of AE was lower than in other studies [22]. Not a single life-threatening adverse event was reported. The most frequent severe adverse event was peripheral neuropathy, mostly related to the use of linezolid at 600 mg per day. An even higher dose of linezolid, a component of a highly effective short all-oral regimen that was recently tested in patients with fluoroquinolone-resistant TB or complicated multi drug resistant TB [28], would require more frequent monitoring, which seems not feasible in our setting with compromised access to care. Future research should prioritize the development of a tolerable short all-oral RR-TB treatment regimen, also effective in patients with fluoroquinolone-resistant TB, and that can be administered regardless of age or pregnancy status.

The majority of our patients were females of child bearing age (60.3%), which contrasts with the higher proportion of males in global TB cohorts. A similar relatively high proportion of female patients was reported in neighboring Pakistan [10, 21]. This may be explained by the traditional role of women, who spend more time indoors and are the main caregivers of sick family members. Overall, one fifth of patients was diagnosed through systematic and repetitive screening among household contacts. Such approach is particularly important in contexts with a high TB burden among children and women, especially when cultural and gender barriers result in diagnostic delay [29, 30]. Among 112 patients on treatment, 16 (14.3%) were children (< 15 years old). Globally 3% of all RR/MDR-TB cases are children, as only 3–4% of children with RR-TB are being diagnosed and put on treatment [31]. Our cohort displayed a five-fold higher proportion of children, which may be explained by systematic household contact tracing [32]. In other studies, between 0.2% -11.2% of children were diagnosed with RR-TB disease after RR-TB household contact screening [33–39].

The Afghanistan National Tuberculosis Control Programme has successfully decentralized drug-susceptible TB care since 2005 [40, 41], but decentralization of RR-TB care is still in progress. Of 146 patients diagnosed with RR-TB, 60.9% came from outside Kandahar as RR-TB care was not available in their place of origin. We observed that a high proportion of those eligible refused RR-TB treatment. Others died before starting treatment. Reasons for refusal need to be explored further. The high frequency of pre-treatment attrition is a major public health problem as it contributes to continuous RR-TB transmission in communities and results in a high mortality among those not started on treatment. Decentralization of RR-TB care reduces the treatment gap and increases retention in care [42]. Integration of RR-TB care into primary health care services in conflict settings was described as feasible [43] and could be a possible approach for the country in the future. Furthermore, community based models of care, including administration of the injectable by a family member, have been described and could be adapted to the context of Afghanistan, if security allows it [44].

This is the first RR-TB study from Afghanistan. Our programme data show the reality of RR-TB care in Kandahar. However, this study also has several limitations. Due to a small sample size we were able to identify only those predictors that were strongly correlated with having an unfavorable outcome, while weaker correlations may have been missed. This retrospective study relied on routinely collected data, which was not complete for all the variables. Probably minor adverse events were not systematically encoded in the electronic medical files. Furthermore, baseline DST results were missing for a substantial proportion of patients, which reflects the reality of most RR-TB programmes today. The effect of initial resistance on treatment outcomes should be addressed by future research. This study was not designed to assess reasons of high pre-treatment attrition. Qualitative research studies should be planned to gather more information on factors that could improve patient-centeredness. Our findings reflect the reality of Kandahar, but do not show the bigger picture of the RR-TB epidemic in Afghanistan. Studies focusing on analysis of DR-TB care outcomes in specific age subgroups or those investigating the impact of social and economic factors need to be considered.

## Conclusion

The translation and adaptation of formal TB treatment guidelines into practice is not straightforward in complex war-torn settings such as Kandahar. We have shown that a patient–centered approach resulted in the diagnosis of RR-TB in vulnerable groups, such as children and woman, and assured that patients received the regimen that suited them best. Even though criteria for the choice of treatment regimen were not strictly implemented, outcomes of both the STR and longer regimen were good. WHO now encourages countries to pilot the use of all-oral RR-TB regimens under operational research conditions. In this setting the baseline resistance pattern, demographic characteristics of the population, and barriers to adverse event monitoring will need to be accounted for when designing such al-oral regimen.
