# Correction: Translating drug resistant tuberculosis treatment guidelines to reality in war-torn Kandahar, Afghanistan: A retrospective cohort study

**DOI:** 10.1371/journal.pone.0299913

**Published:** 2024-02-28

**Authors:** Anita Mesic, Waliullah H. Khan, Annick Lenglet, Lutgarde Lynen, Sadiqqulah Ishaq, Ei Hnin Hnin Phyu, Htay Thet Mar, Anthony Oraegbu, Mohammad Khaled Seddiq, Hashim Khan Amirzada, Jena Fernhout, Charity Kamau, Cono Ariti, Diana Gomez, Tom Decroo

There are errors in the Funding and Competing Interests statements. The correct statements are:

**Funding:** Médecins Sans Frontières (MSF) provided support in the form of salaries for AM, WHK, AL, SI, EHHP, HTM, AO, JF, CK, and DG. The specific roles of these authors are articulated in the ‘author contributions’ section. MSF programmatic funding covered all costs of the study, including the publication fee. The funder had a role in study design, data collection and analysis, decision to publish and preparation of the manuscript. This is indicated by the affiliation of the co-authors and their role in the study and publication.

**Competing Interests:** The authors have read the journal’s policy and have the following competing interests to declare: Médecins Sans Frontières (MSF) provided support in the form of salaries for AM, WHK, AL, SI, EHHP, HTM, AO, JF, CK, and DG. This does not alter our adherence to *PLOS ONE* policies on sharing data and materials. There are no patents, products in development, or marketed products associated with this research to declare.
